# Mechanical disturbances applied by motorized ankle foot orthosis to adapt ankle muscles activation—A validation study

**DOI:** 10.3389/fbioe.2023.1079027

**Published:** 2023-03-16

**Authors:** Guillermo Asín-Prieto, Filipe Oliveira Barroso, Aitor Martínez-Expósito, Eloy Urendes, Jose Gonzalez-Vargas, Juan C. Moreno

**Affiliations:** ^1^ Neural Rehabilitation Group, Cajal Institute, Spanish National Research Council (CSIC), Madrid, Spain; ^2^ Gogoa Mobility Robots, Abadiño, Spain; ^3^ Universidad Autónoma de Madrid, Madrid, Spain; ^4^ Departamento de Tecnologías de la Información, Escuela Politécnica Superior, Escuela Politécnica Superior, Universidad San Pablo-CEU, CEU Universities, Madrid, Spain; ^5^ Global Research, Ottobock SE & Co. KGaA, Duderstadt, Germany

**Keywords:** robotic devices, motorized ankle foot orthosis, MAFO, electromyography, adaptation

## Abstract

**Background:** Reduced function of ankle muscles usually leads to impaired gait. Motorized ankle foot orthoses (MAFOs) have shown potential to improve neuromuscular control and increase volitional engagement of ankle muscles. In this study, we hypothesize that specific disturbances (adaptive resistance-based perturbations to the planned trajectory) applied by a MAFO can be used to adapt the activity of ankle muscles. The first goal of this exploratory study was to test and validate two different ankle disturbances based on plantarflexion and dorsiflexion resistance while training in standing still position. The second goal was to assess neuromuscular adaptation to these approaches, namely, in terms of individual muscle activation and co-activation of antagonists.

**Methods:** Two ankle disturbances were tested in ten healthy subjects. For each subject, the dominant ankle followed a target trajectory while the contralateral leg was standing still: a) dorsiflexion torque during the first part of the trajectory (Stance Correlate disturbance—StC), and b) plantarflexion torque during the second part of the trajectory (Swing Correlate disturbance—SwC). Electromyography was recorded from the tibialis anterior (TAnt) and gastrocnemius medialis (GMed) during MAFO and treadmill (baseline) trials.

**Results:** GMed (plantarflexor muscle) activation decreased in all subjects during the application of StC, indicating that dorsiflexion torque did not enhance GMed activity. On the other hand, TAnt (dorsiflexor muscle) activation increased when SwC was applied, indicating that plantarflexion torque succeeded in enhancing TAnt activation. For each disturbance paradigm, there was no antagonist muscle co-activation accompanying agonist muscle activity changes.

**Conclusion:** We successfully tested novel ankle disturbance approaches that can be explored as potential resistance strategies in MAFO training. Results from SwC training warrant further investigation to promote specific motor recovery and learning of dorsiflexion in neural-impaired patients. This training can potentially be beneficial during intermediate phases of rehabilitation prior to overground exoskeleton-assisted walking. Decreased activation of GMed during StC might be attributed to the unloaded body weight in the ipsilateral side, which typically decreases activation of anti-gravity muscles. Neural adaptation to StC needs to be studied thoroughly in different postures in futures studies.

## 1 Introduction

Neural impairments usually lead to inappropriate muscle activation and, consequently, to impaired motor function (Safavynia et al., 2011; Cheung et al., 2012). Disrupted walking function due to impaired dorsi-plantar flexion is a common disability in patients with stroke (Zehr, 2011; Barroso et al., 2017), spinal cord injury (Van Der Salm et al., 2005; Ditunno and Scivoletto, 2009), cerebral palsy (Conner et al., 2020) and other neurological disorders.

Repetitive and intensive task-specific training (*e.g.*, gait training) may drive beneficial neuroplasticity and, thus, enhance functional recovery (Stampacchia et al., 2016; Barroso et al., 2019b). In this regard, robotic therapy has been gradually used in motor rehabilitation practice aiming at promoting gait recovery in patients who suffered neural impairments such as stroke and spinal cord injury (Moreno et al., 2013). Robotic devices allow multiple, intensive and longer training sessions, apart from promoting active engagement of the user (Barroso et al., 2019b), which are core principles of motor learning (Belda-Lois et al., 2011; Winstein et al., 2014; Conner et al., 2021) and that might help some of these patients to restore or improve gait patterns.

Given that the ankle joint is crucial to achieve adequate walking function and that its rehabilitation may ultimately improve gait performance, several motorized ankle foot orthoses (MAFOs) have been developed in the past 2 decades (Moltedo et al., 2018). Training with MAFOs has led to clinical benefits, including improvement of gait independence, toe clearance strategy, as well as reduction of compensatory movements of hemiparetic stroke patients (Pongpipatpaiboon et al., 2018; Yeung et al., 2018; Shi et al., 2019), spinal cord injury patients Arazpour et al. (2013) and children with cerebral palsy Conner et al. (2021).

Currently available controllers for MAFOs vary from position controlled trajectories based on healthy patterns to force and impedance controllers (Shi et al., 2019), and myoelectric-based controllers (Takahashi et al., 2015; Ceseracciu et al., 2017). Development of novel MAFO control strategies might take into account that improved performance and cortical reorganization can be potentiated through active neuromuscular engagement. In this regard, adaptive plantarflexion resistance during the propulsive phase of gait led to increased strength and improved neuromuscular control in cerebral palsy individuals following exoskeleton training intervention (Conner et al., 2020). Recently, we have also presented a new robotic therapy protocol using a non-ambulatory exoskeleton that perturbed the ankle joint based on tacit learning control - a symbiotic control strategy inspired by biomimetic mechanisms (Asín-Prieto et al., 2020). Results showed improvements with respect to task performance and motor outcomes in both healthy individuals and a post-stroke patient.

In this study, we hypothesize that adaptation of ankle muscles activity can be achieved through customized adaptive resistance using our MAFO platform in standing still position. Demonstration of our hypothesis will add more evidence that our MAFO platform, which was designed to be used during intermediate phases of rehabilitation (between joint mobilization and gait training), can be further explored to achieve targeted neuromuscular rehabilitation in neurological conditions such as stroke and spinal cord injury. Therefore, the first goal of this exploratory study was to test and validate two different ankle disturbances (perturbations to the planned trajectory) based on plantarflexion and dorsiflexion resistance during MAFO training. The second goal of the study was to assess muscle adaptation to this MAFO training.

Two disturbances were tested in ten healthy subjects while the dominant ankle followed a target trajectory and the contralateral leg was standing still: a) dorsiflexion torque (disturbance in upward direction) during the first part of the trajectory (Stance Correlate disturbance - StC), and b) plantarflexion torque (disturbance in downward direction) during the second part of the trajectory (Swing Correlate disturbance—SwC) (Conner et al., 2020). Our first hypothesis was that applying StC disturbance (during stance) would selectively enhance ankle plantarflexor (gastrocnemius medialis, GMed) activation while ankle dorsiflexor (tibialis anterior, TAnt) activation would remain unchanged. Our second hypothesis was that applying SwC disturbance (during swing) would selectively enhance TAnt activation while GMed activation would remain unchanged.

These novel paradigms may set some bases for future rehabilitation strategies using MAFOs. Furthermore, they can potentially be beneficial during intermediate phases of rehabilitation, prior to overground exoskeleton-assisted walking.

## 2 Materials and methods

### 2.1 Subjects

Ten healthy volunteers (five females and five males; age: 26.10 ± 3.81 years; height: 171.30 ± 9.75 cm; weight: 66.51 ± 8.13 kg), with no neurological injuries, volunteered to participate in this study. Participants were informed about the procedures and possible discomfort associated with the experiments. After that, they signed an informed consent to participate. All procedures were conducted in accordance with the Declaration of Helsinki and approved by the Bioethical Subcommittee of the Ethics Committee of CSIC (Spanish National Research Council, reference 008/2016).

### 2.2 Experimental platform

**FIGURE 1:**
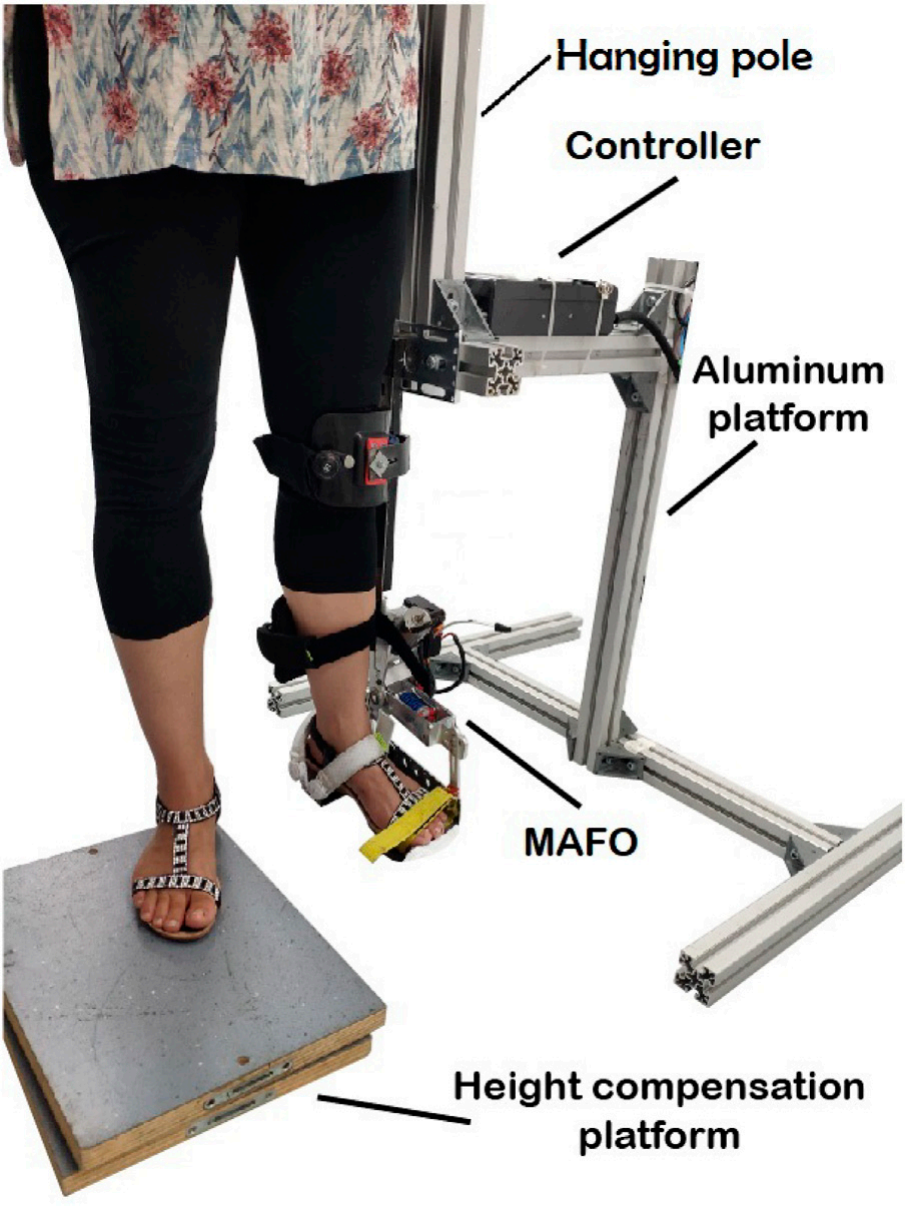
Volunteer following a torque profile trajectory with the ankle during MAFO training.

The first part of the experiment was performed using an experimental platform that is able to control and add disturbances (perturbations to the target trajectory) to ankle joint torque during a standing still position (see Figure 1) and that has been previously described in detail in Asín-Prieto et al. (2020). In this previous study, we used the experimental platform to test in a post-stroke patient and in healthy subjects a new robotic therapy based on tacit learning control, with participants being sat during each trial. The rationale for applying disturbances to the target trajectory builds on previous research showing that adaptive ankle resistance implemented with wearable exoskeletons leads to improved neuromuscular control and increased volitional engagement of the ankle muscles (Conner et al., 2020).

**FIGURE 2:**
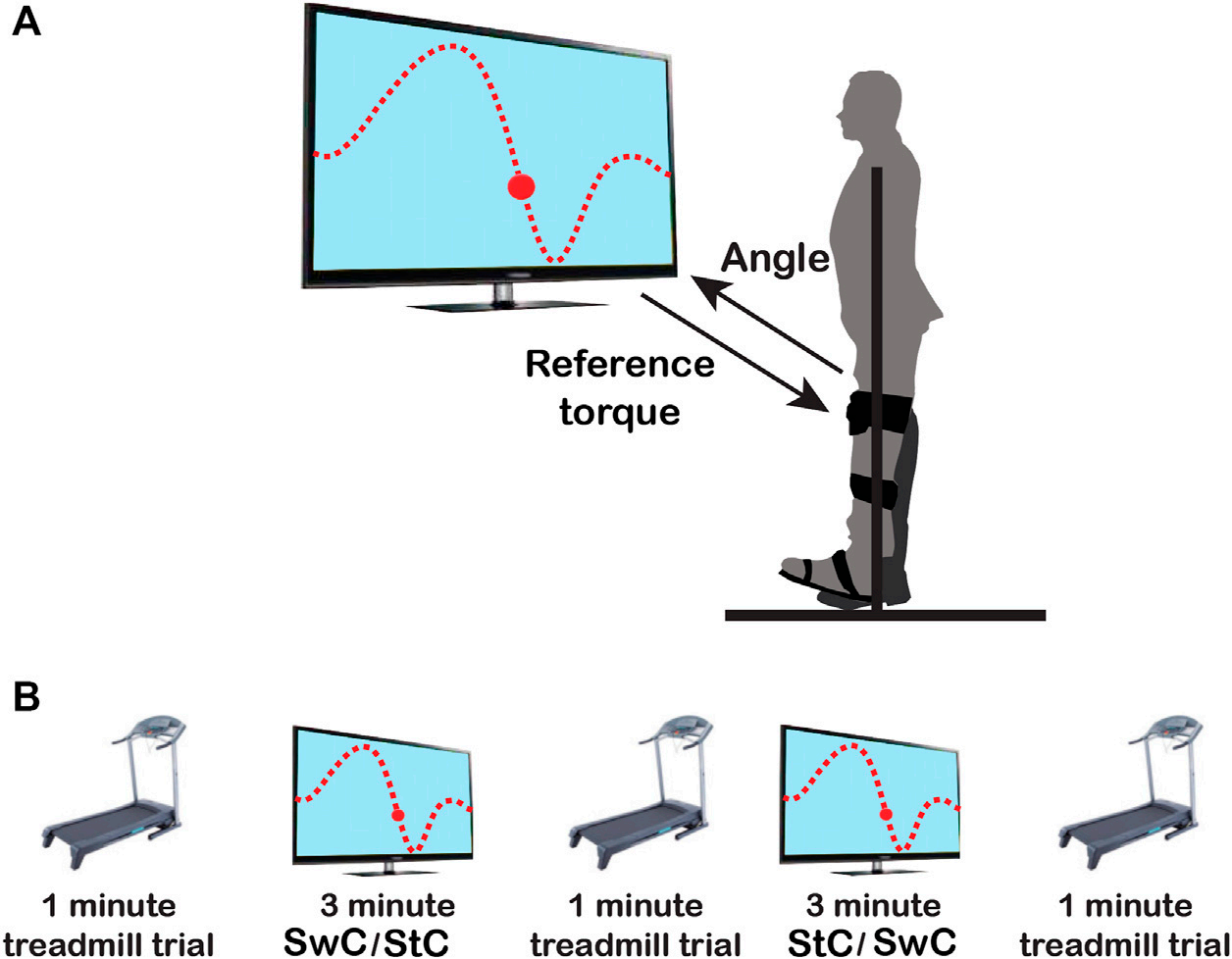
Schematics of the experimental protocol. **(A)** The upper panel shows the experimental setup. Subjects’ dominant ankle had to follow the up-downwards movement presented on the screen as a dotted line, mimicking the normal ankle pattern in the sagittal plane during gait. **(B)** The lower panel shows the order of trials along the session: 1) 1-min treadmill trial; 2) StC or SwC trial, for three minutes; 3) 1-min treadmill trial; 4) SwC or StC trial, for three minutes; and 5) last 1-min treadmill trial. Periods of five minutes of rest between sessions were included to avoid muscle fatigue.

The visual paradigm was implemented as a video game consisting of a character (ball) and a line to be followed by the subject (see Figure 2A and Additional file 1), while the MAFO exerted a controlled torque. Volunteers had to support their body weight by hanging their ipsilateral hand on the hanging pole rigidly attached to the aluminium structure, thus avoiding leaning towards the structure.

### 2.3 MAFO-based exercise paradigms

Each participant executed two different MAFO-based exercise paradigms while the dominant (ipsilateral) ankle followed a target trajectory and the contralateral leg was standing still (see Additional file 1): a) dorsiflexion torque (upward disturbance against target trajectory) during the first part of the trajectory (Stance Correlate—StC—disturbance), and b) plantarflexion torque (downward disturbance against target trajectory) during the second part of the trajectory (Swing Correlate—SwC—disturbance).

**FIGURE 3:**
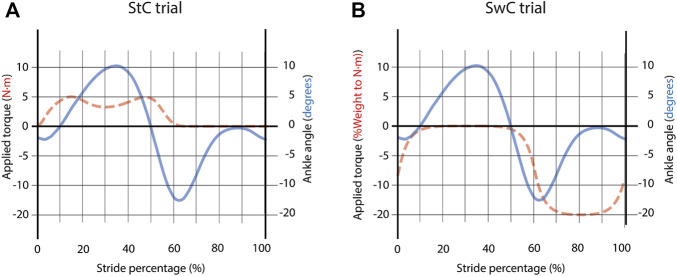
**(A)** Torque profile and trajectory to follow for StC trials. Dorsiflexion torque (disturbance in upward direction) had the shape of ground reaction force to mimic this torque during StC. **(B)** Torque profile and trajectory to follow for SwC trials. As there is no ground reaction force during swing in normal gait (foot is not in touch with the ground), a plantarflexion torque (disturbance in downward direction) proportional to the weight of the subject was applied during SwC. (StC—Stance Correlate disturbance. SwC—Swing Correlate disturbance).

The StC trial consisted of a total of 86 cycles. For the full trial, a dorsiflexion torque with a maximum of 5 N⋅m (technical limitation of the MAFO for dorsiflexion) was applied from 0% to 60% of each cycle duration, to force each subject to perform plantarflexion (and thus enhance GMed activation) in order to reach the prescribed trajectory (see Figure 3A). Dorsiflexion torque (disturbance in upward direction) had the shape of ground reaction force to mimic this torque during StC.

The SwC trial also consisted of a total of 86 cycles. For the first 70 cycles, a plantarflexion torque proportional to the weight of the subject (20% of subject’s weight, with a maximum limit of 17 N⋅m—technical limitation of the MAFO for plantarflexion), was applied from 60% to 100% of each cycle duration (as a correlate of swing phase) to force dorsiflexion (and thus enhance TAnt activation) in order to reach the prescribed trajectory (see Figure 3B). As there is no ground reaction force during swing in normal gait (foot is not in touch with the ground), a plantarflexion torque (disturbance in downward direction) proportional to the weight of the subject was applied during SwC. At the 71st step, the torque was removed in order to study the adaptation effect to the SwC strategy for 16 more cycles (from a total of 86 cycles).

### 2.4 Experimental protocol and data collection

For each participant, the training session (depicted in Figure 2B) consisted of two MAFO-based exercise trials (three minutes each one) and three treadmill trials (1-min each) at the beginning, between MAFO trials, and at the end of the session. The order of MAFO-based trials was randomized (first StC and then SwC, or *vice versa*) to avoid the effect of training order. Periods of five minutes of rest between trials were included to avoid muscle fatigue. During the treadmill trials, participants were asked to maintain a constant walking cadence, set by an auditory metronome (Barroso et al., 2013), of approximately 30 strides/minute (slow walking) (Tudor-Locke et al., 2011; Paul et al., 2016).

During the two different MAFO trials, the user had to follow, *via* dorsi-plantar flexion, the up-downwards movement presented on the screen as a dotted line, mimicking the normal ankle pattern in the sagittal plane during gait (Winter 1991) for a cadence of approximately 30 cycles/minute.

Participants were placed on a height compensation platform (to leave free range of motion for the instrumented ankle). For each subject, the dominant leg was placed inside the cuffs of the non-ambulatory MAFO, and the cuffs were attached to the following locations: foot tip, instep, heel, shin, and just below the knee (see Additional file 2).

For the full session, an electromyography (EMG) amplifier (Quattrocento, OT Bioelettronica, Torino, Italy) was used to record muscle activity of two muscles acting at the dominant ankle joint: TAnt and GMed. EMG signals were recorded with an acquisition frequency of 5,120 Hz (Kaczmarek et al., 2019), overall gain of 150 and were electronically bandpass filtered (10–4,400 Hz). SENIAM (European concerted action focused on recommendations and proposition of standards for surface electromyography - sEMG) guidelines for sEMG recording (cleaning the skin area where the electrodes would be placed with alcohol to reduce impedance) and muscle identification were followed (Hermens et al., 1999). After that, sEMG bipolar electrodes (Ag-AgCl, Ambu Neuroline 720, Ambu, Ballerup, Denmark) were placed with 2 cm inter-electrode distance over the belly of the two muscles. After that, electrodes and cables were wrapped with bandages. Preliminary tests to check the quality of the signal and proper electrode positioning were also performed. The EMG reference was placed on the patella of the instrumented leg.

During the MAFO trials, we also recorded the angular position performed by the ankle at 128 Hz. For the treadmill trials, a footswitch (FSR 406–38 × 83 mm—square, Interlink Electronics, Camarillo, CA, United States) was placed beneath the heel of the dominant leg in order to record heel strike moments during walking (Barroso et al., 2014). Data from the footswitch were used to define heel strike and heel off events, which were used to segment each locomotion stride.

EMG data and ankle joint angle were synchronized using a common trigger signal, which was controlled by the volunteer to start each of the MAFO trials. Data were stored for offline analyses and were analysed with MATLAB R2016a (Mathworks, Natick, MA, United States) and IBM SPSS Statistics 25 software (IBM, Armonk, N.Y. United States).

### 2.5 Data processing

#### 2.5.1 EMG analysis

Raw EMG signals were bandpass filtered offline to remove DC offset and motion artefacts (20–450 Hz, 2nd order Butterworth), demeaned, rectified, and low-pass filtered at 4 Hz (2nd order Butterworth), resulting in the EMG envelopes. Based on cycle segmentation information (from the footswitch and from the MAFO), EMG envelopes were then resampled at each 1% of the cycle, so that each cycle consisted of 100 data points (Barroso et al., 2014).

To assess TAnt and GMed activity across trials for each subject, we divided the analysis of EMG data into the two main gait cycle phases: stance and swing. This was done assuming that stance corresponded to 0%–60% of cycle duration and swing to 60%–100% of cycle duration (Winter 1991). For each subject, we then integrated the EMG envelopes activity (area under the EMG envelopes) during each phase and trial, which allowed us to compare muscle activity across trials (Barroso et al., 2019a). For each participant, muscle and cycle phase, integrated activity was normalized by the mean of the integrated activity in the first treadmill trial, which was used as a baseline value (Alessandro et al., 2020). Moreover, for the SwC trials, data were divided into three specific phases of the trial, in order to study the effects after applying the disturbances: 1) first 70 cycles, when the torque was applied - called SwC ON; 2) cycles 71–76, corresponding to the first stage of torque removal (to assess the effects of the adaptation in the previous 70 cycles due to the applied torque)—called SwC OFF 1; and 3) cycles 77–86, corresponding to the second stage of torque removal (to study the evolution of this adaptation) - called SwC OFF 2.

### 2.5.2 Kinematics

For each subject, kinematics data (ankle angle) from MAFO trials were resampled at each 1% of the cycle, so that each cycle consisted of 100 data points. The mean ankle angle exhibited by each participant during SwC training was used to calculate the peak of dorsiflexion during swing. Differences between the mean trajectories performed during the three phases of SwC training (SwC ON, SwC OFF 1 and SwC OFF 2) and the target trajectory were assessed using two criteria: rmax coefficient, which is the maximum of the cross-correlation between two signals; and the lag time, which is the maximum of the cross-correlation function obtained using the MATLAB xcorr function for centered data (option = “coeff”) and the output values as the maximum of the xcorr function (Hug et al., 2011). These two values provided an indication of the similarity of trajectory shapes.

For each participant, MAFO trial and phase (stance and swing), the error (mathematical subtraction) between the maximum angle performed and the maximum target angle were also calculated.

### 2.6 Statistical analysis

The non-negative EMG distributions and kinematics analysed here were inspected for normality using the Shapiro-Wilk test. Since normality was not verified, we applied non-parametric tests. We assessed differences in EMGs for all conditions using Friedman tests of differences among repeated measures, evaluating the size effect with the Bonferroni-adjusted exact *p*-value (**e** *p* from now on) (Eisinga et al., 2017).

To compare the different angular positions (before and after the effect) for the SwC condition and the target angular trajectory, we used the Wilcoxon signed-rank test, evaluating the size effect by Cliff’s $\bar{\delta }$ test.

For either stance or swing, the ankle joint mostly goes from plantarflexed position to dorsiflexed position. In normal gait, the foot is flat (0 degrees) at around 10% of the cycle and then the tibia moves, with dorsiflexion reaching a maximum of 10 degrees as the tibia moves over the ankle joint. On the other hand, swing phase starts with the ankle at maximum plantarflexion and continues with rapid dorsiflexion to allow the clearance of the foot from the ground. Therefore, for each paradigm (SwC and StC) and phase (stance and swing), the error between maximum performed angle and maximum target angle was correlated with integrated muscle activity using Pearson’s correlation coefficient, to investigate if differences in ankle angles could be explained by changes in muscle activity.

Statistical significance was set by a *p*-value of 0.05.

## 3 Results

### 3.1 Treadmill trials

**FIGURE 4:**
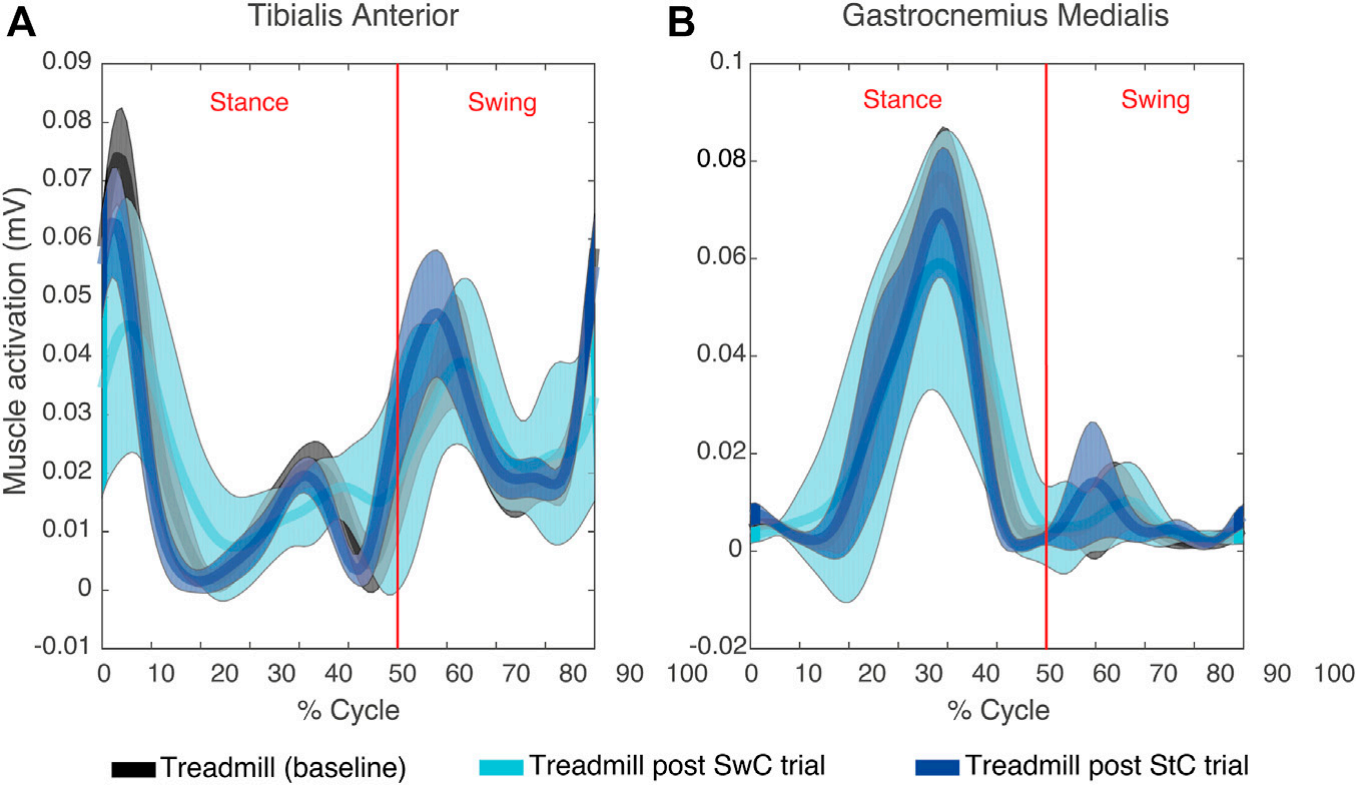
Mean (thick lines) and standard deviation (shaded areas) of EMG envelopes from the tibialis anterior **(A)** and gastrocnemius medialis **(B)** of a representative subject (ID 09) during the three treadmill trials of the experimental session. Vertical red lines divide stance and swing phases.

Figure 4 illustrates the mean EMG envelopes of a representative subject (ID 09) during treadmill trials. These trials were performed at very low speed (approximately 30 strides/minute). Therefore, muscle activity was very low. For TAnt, most of the activation happened during initial loading (0%–12% of gait cycle), initial swing (62%–75% of gait cycle) and midswing (75%–87% of gait cycle). For GMed, most of the activation was observed in terminal stance (30%–50% of gait cycle). In general, all subjects presented very similar activation profiles for TAnt and GMed when comparing the three treadmill trials.

### 3.2 Stance correlate (StC) trials

**FIGURE 5:**
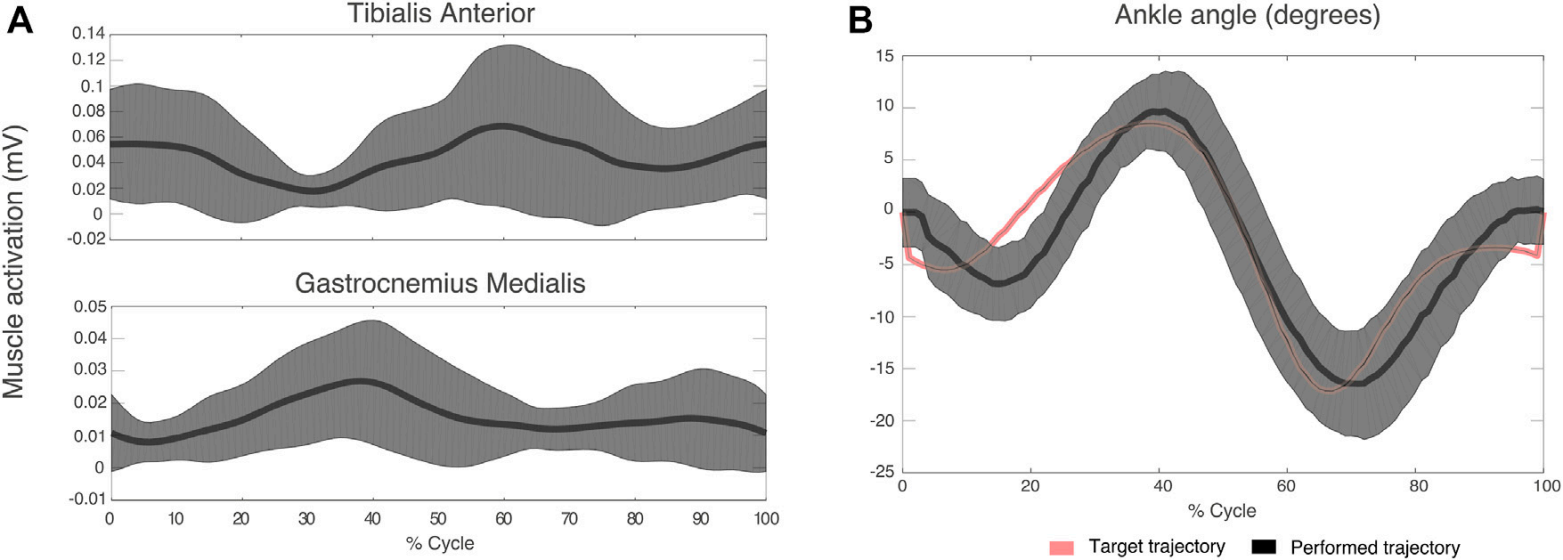
**(A)** Mean (thick lines) and standard deviation (shaded areas) of EMG envelopes from the tibialis anterior and gastrocnemius medialis of a representative subject (ID 09) during the StC trial. **(B)** Target and performed ankle angle during the StC trial for the same subject as in **(A)**. Positive angle indicates dorsiflexion direction and negative angle indicates plantaflexion.

Figure 5A shows mean TAnt and GMed EMG envelopes of a representative subject during the StC trial. Figure 5B represents target ankle angle, as well as the ankle angle performed by the same representative subject during the StC trial.

**FIGURE 6:**
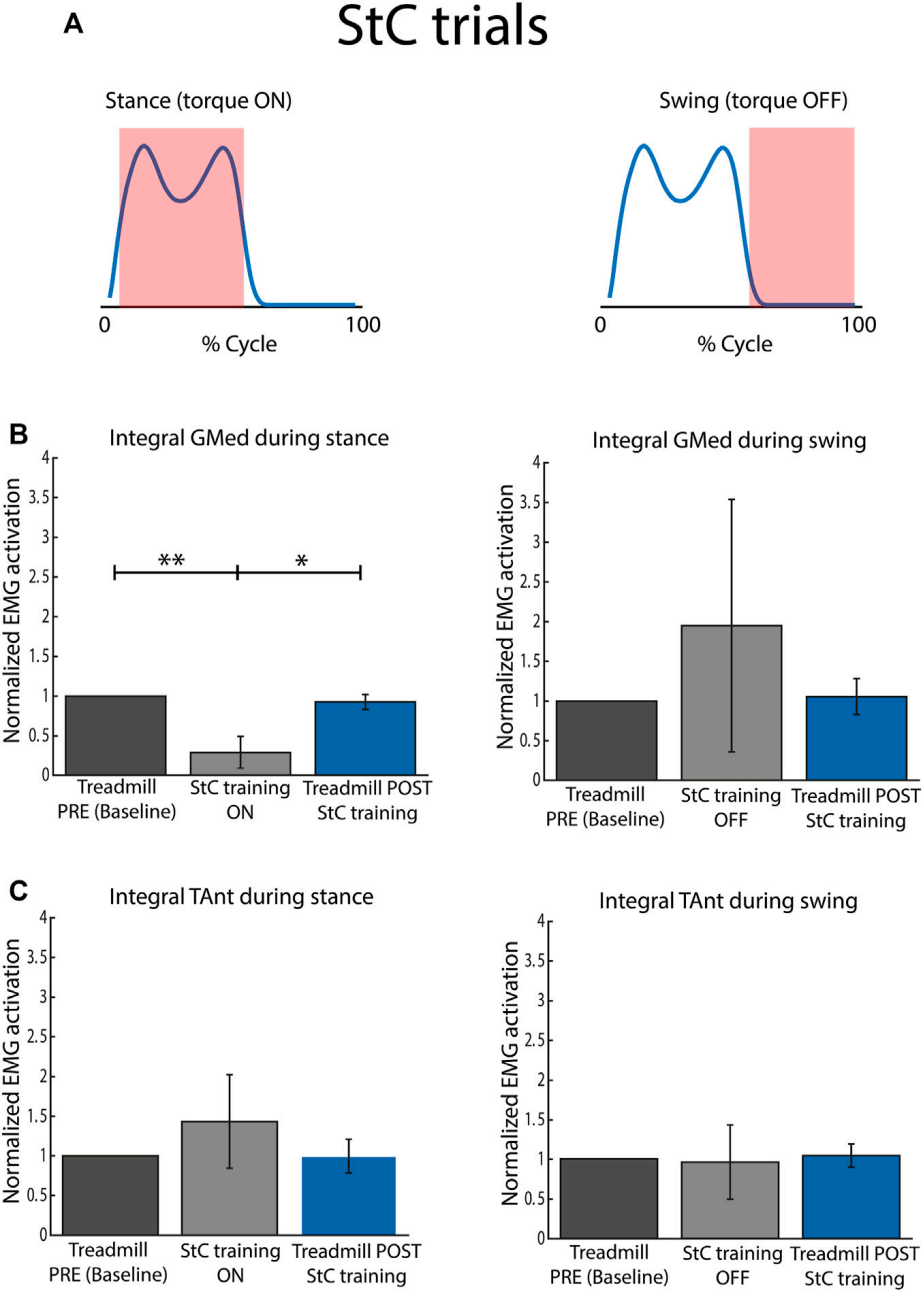
Normalized EMG activation across subjects for StC and treadmill (PRE and POST StC training) trials. **(A)** Torque profile delivered during stance and swing (no disturbances) under StC disturbances. **(B)** Activation of GMed during stance (left panel) and swing (right panel), normalized to the baseline, *i.e.*, Treadmill PRE. **(C)** Activation of TAnt during stance (left panel) and swing (right panel), normalized to the baseline, *i.e.*, Treadmill PRE. Bars represent mean and standard deviation of the group. *p* < 0.05*; *p* < 0.01**.

Figure 6A represents the torque profile delivered during stance and swing for StC trials. In particular, there was a dorsiflexion torque (upwards) applied from 0% to 60% of cycle duration (stance), while there was no torque applied during 60%–100% of cycle duration (swing).

Figure 6B represents mean integrated EMG activity of GMed during StC training for the whole group. During the stance phase, integrated GMed activity was significantly lower during StC trials than during baseline (*χ* ^2^ = 15.80; *p* < 0.05; **e** *p* = 0.00 for Treadmill PRE vs. StC; and **e** *p* = 0.01 for StC vs. Treadmill POST). For the swing phase, there were no statistically significant differences between GMed activity during StC trials and treadmill trials.

Figure 6C represents mean integrated EMG activity of TAnt during StC training for the whole group. There were no significant differences between conditions (StC and treadmill trials) for each of the cycle phases (stance and swing).

**FIGURE 7:**
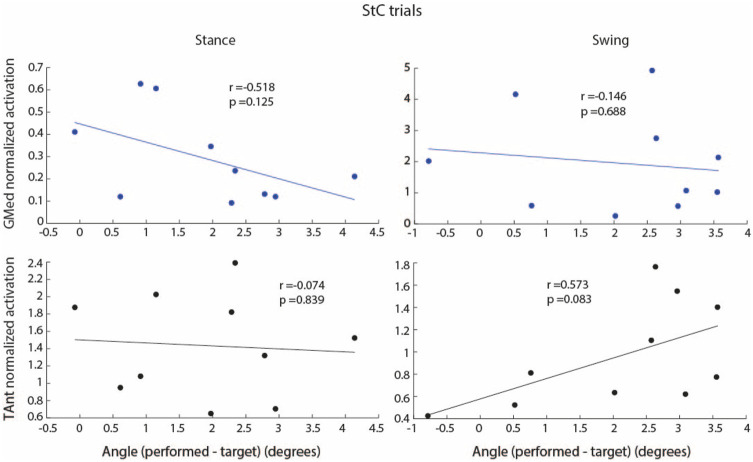
Correlation between integrated EMG activity of GMed (blue dots and regression lines) and TAnt (black dots and regression lines) and the error (mathematical subtraction) between maximum performed ankle angle and maximum target angle for stance and swing phases during StC trials. Each dot represents each subject.

Figure 7 shows the correlation between integrated EMG activity of ankle muscles (GMed and TAnt) and the error between maximum dorsiflexion angle performed and maximum target angle, for stance and swing, during StC trials. No significant correlations between individual EMG activity of ankle muscles and the maximum ankle angle performed were found either when StC torque was applied (stance) or when StC torque was not applied (swing).

### 3.3 Swing correlate (SwC) trials

**FIGURE 8:**
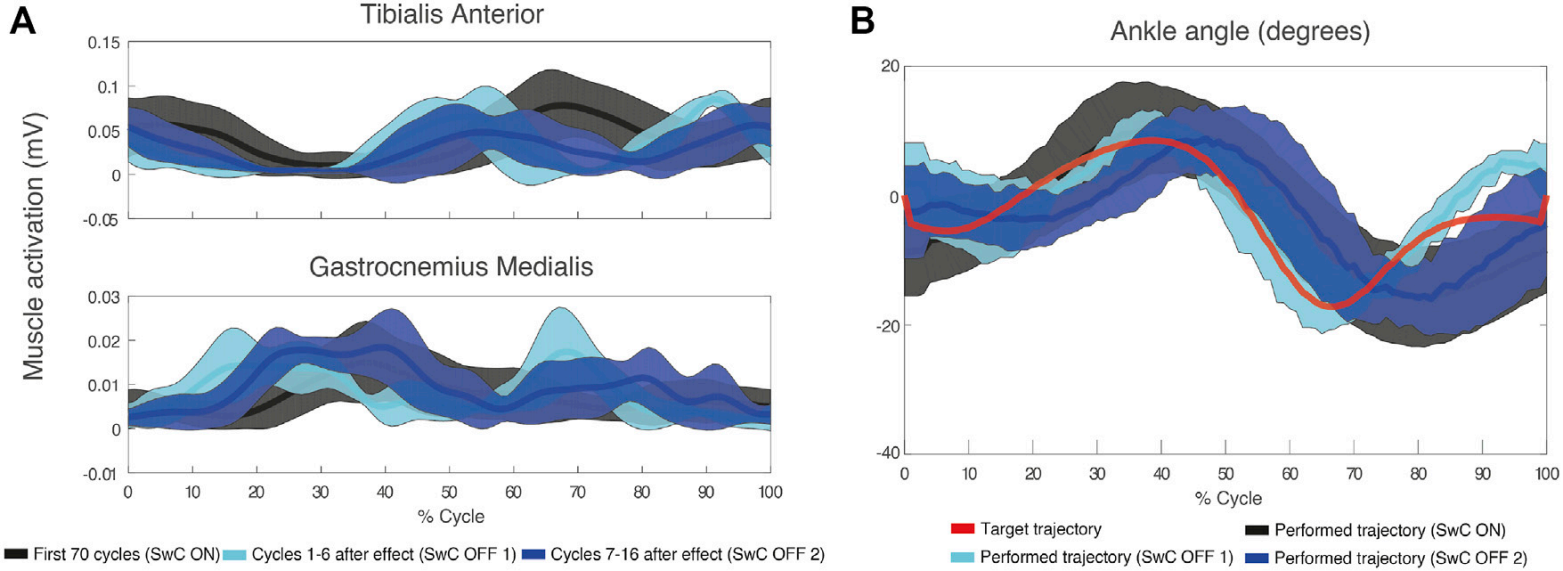
**(A)** Mean (thick lines) and standard deviation (shaded areas) of EMG envelopes from the tibialis anterior and gastrocnemius medialis of a representative subject (ID 09) during SwC trial. **(B)** Target and performed ankle trajectory during the three specific phases of SwC trials: SwC ON (first 70 cycles), SwC OFF 1 (first stage after removing SwC torque), and SwC OFF 2 (second stage after removing SwC torque). Positive angles indicate dorsiflexion and negative angles indicate plantarflexion.

Figure 8A shows TAnt and GMed EMG envelopes obtained from a representative subject during SwC trials. Data were divided into three specific phases of the trials: SwC ON (first 70 cycles), SwC OFF 1 (first stage after removing SwC torque), and SwC OFF 2 (second stage after removing SwC torque). Qualitatively, EMG profiles from TAnt and GMed were different for each of the three SwC phases.

**FIGURE 9:**
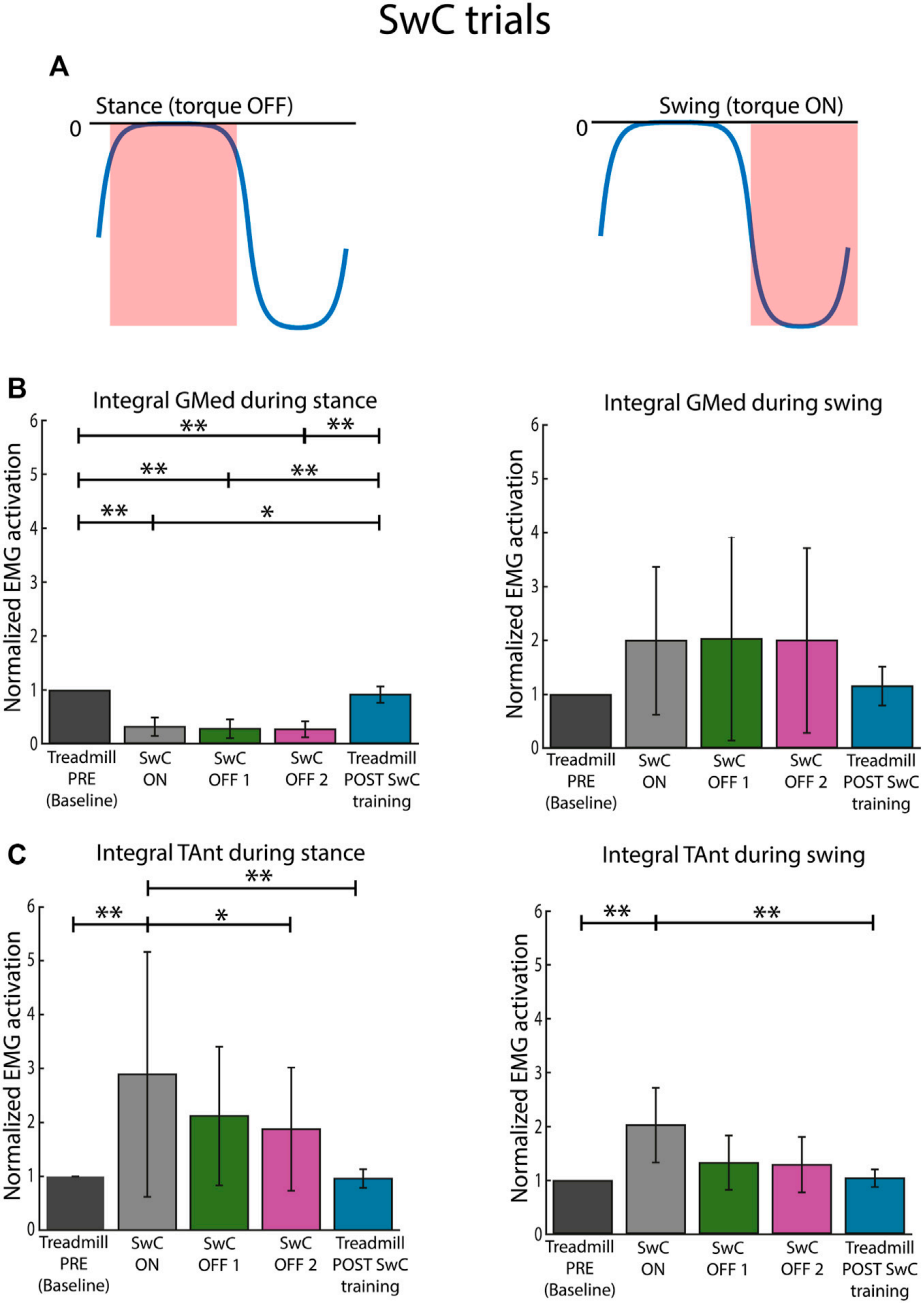
Normalized EMG activation across subjects for SwC and treadmill (PRE and POST SwC training) trials. **(A)** Torque profile delivered during stance (no disturbances) and swing under SwC disturbances. **(B)** Activation of GMed during stance (left panel) and swing (right panel), normalized to the baseline, *i.e.*, Treadmill PRE. **(C)** Activation of TAnt during stance (left panel) and swing (right panel), normalized to the baseline, *i.e.*, Treadmill PRE. SwC ON corresponds to the first 70 training cycles with the torque. SwC OFF 1 corresponds to training cycles 1–6 without the SwC torque. SwC OFF 2 corresponds to training cycles 7–16 without the SwC torque. Bars represent mean and standard deviation of the group. *p* < 0.05*; *p* < 0.01**.

Figure 9A represents the torque profiles during stance and swing for SwC trials. In particular, there was no torque applied during 0%–60% of cycle duration (stance), but there was a plantarflexion torque (downwards) applied from 60% to 100% of cycle duration (swing).

Figure 9B represents mean integrated EMG activity of GMed during SwC training for the whole group. Friedman test rendered statistical changes only for stance phase (*χ* ^2^ = 30.64; p < 0.05; **e** *p* = 0.00 for treadmill baseline vs. SwC ON; **e** *p* = 0.00 for treadmill baseline vs. SwC OFF 1; **e** *p* = 0.00 for treadmill baseline vs. SwC OFF 2; **e** *p* = 0.03 for SwC ON vs. treadmill POST SwC; **e** *p* = 0.01 for SwC OFF 1 vs. treadmill POST SwC; and **e** *p* = 0.00 for SwC OFF 2 vs. treadmill POST SwC). GMed EMG activity was significantly reduced in SwC training trials compared to treadmill trials during stance phase. For all conditions, GMed EMG activity was similar when comparing baseline and treadmill POST SwC trials.

Figure 9C represents mean integrated EMG activity of TAnt during SwC training for the whole group. The results of Friedman test for TAnt rendered significant differences both for stance (*χ* ^2^ = 21.84; p < 0.05; **e** *p* = 0.00 for treadmill baseline vs. SwC ON; **e** *p* = 0.02 for SwC ON vs. SwC OFF 2; and **e** *p* = 0.00 for SwC ON vs. treadmill POST SwC) and swing (*χ* ^2^ = 18.64; p < 0.05; **e** *p* = 0.00 for treadmill baseline vs. SwC ON; and **e** *p* = 0.00 for SwC ON vs. treadmill POST SwC). During stance, TAnt presented significantly increased activity in SwC ON trials compared to baseline, SwC OFF 2 and treadmill POST SwC trials. During swing, TAnt presented significantly increased activity in SwC ON trials compared to baseline and treadmill POST SwC trials. Although mean TAnt activity when SwC was removed (SwC OFF 1 and SwC OFF 2) was on average lower than when the SwC was ON, differences were not statistically significant. For all conditions, TAnt EMG activity was similar when comparing baseline and treadmill POST SwC trials.

Figure 8B represents target ankle angle, as well as the ankle angle performed by the same representative subject from Figure 8A, for the three SwC phases. For this subject, there was an enhanced dorsiflexion during swing immediately after removing the effect (SwC OFF 1), which reverted to baseline values 7–16 cycles after (SwC OFF 2) (SwC ON shaded area is superimposed with SwC OFF 2 shaded area during swing).

**FIGURE 10:**
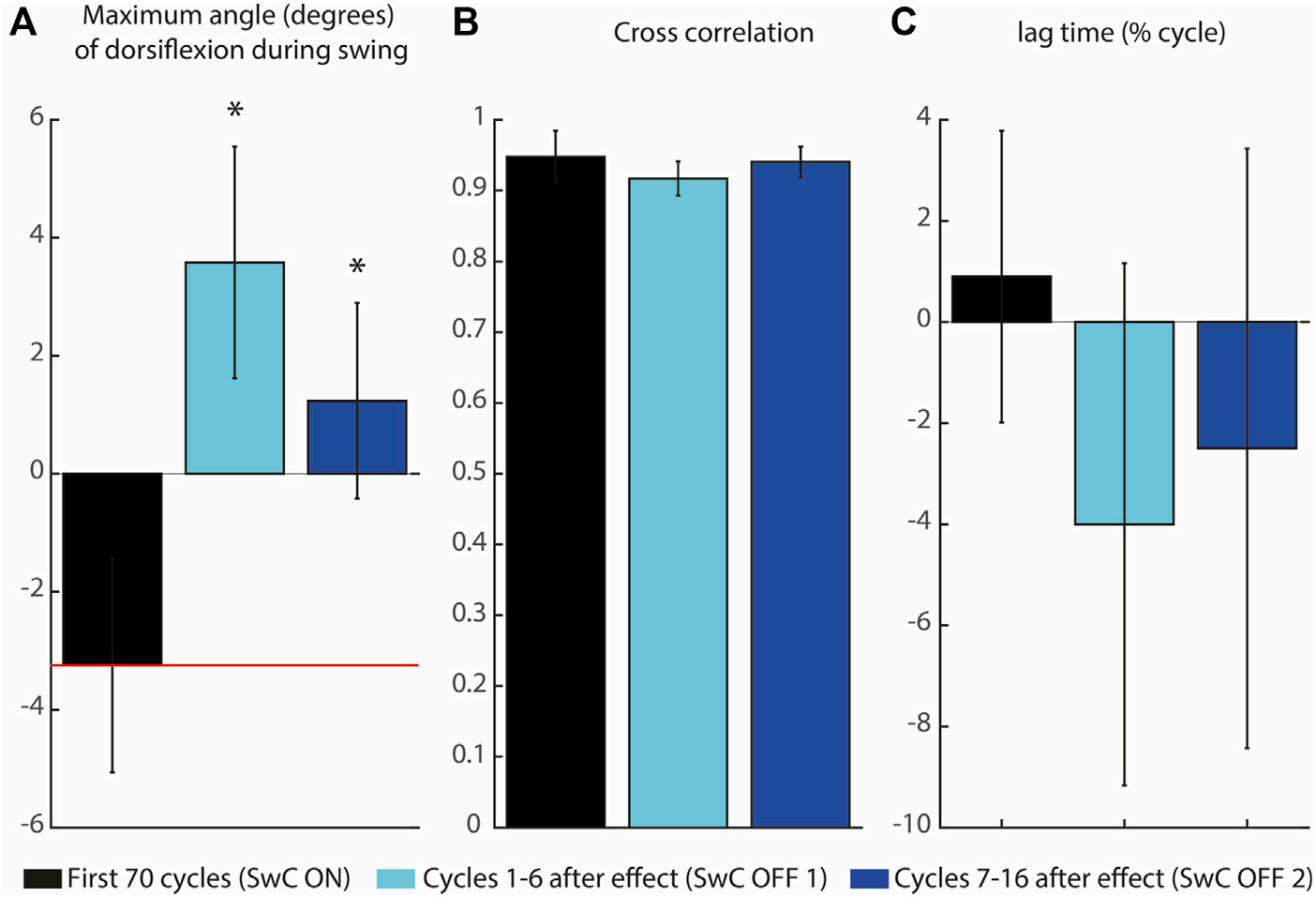
Maximum angle of dorsiflexion during swing and cross-correlation of the three SwC conditions. **(A)** Maximum angle of dorsiflexion during swing. The horizontal red line represents the target peak of dorsiflexion during swing. **(B)** Cross-correlation coefficients between ankle angle trajectories for the three SwC conditions (SwC ON, SwC OFF1 and SwC OFF 2) and the target trajectory. **(C)** Lag time of maximum cross-correlation between the three SwC conditions and target trajectory. Negative lag time means that the trajectory was performed earlier in the cycle relative to the target trajectory. *p* < 0.05*.

Subjects learned to adapt to the target ankle trajectory when SwC torque was applied (swing phase), as represented in Figure 10. Peaks of dorsiflexion during swing phase obtained in SwC trials are represented in Figure 10A. Wilcoxon test between target angle (median = −3.35 degrees) and SwC ON trials (median = −2.80 degrees) showed no significant differences between conditions. On the other hand, when SwC torque was removed, there were increased peaks of dorsiflexion in both SwC OFF 1 (median = 3.75 degrees; Z = −2.80; *p* < 0.05; Cliff’s $\bar{\delta }$ = −1) and SwC OFF 2 (median = 0.94 degrees; Z = −2.80; *p* < 0.05; Cliff’s $\bar{\delta }$ = −1) compared to target angle. On the other hand, cross-correlations depicted in Figure 10B show high correlation of ankle angle compared to target angle, although the timing of maximum correlation indicated anticipation of trajectory (mean lag time = −4.00%, std = 5.16% for SwC OFF 1; mean lag time = −2.5%, std = 5.93% for SwC OFF 2) when disturbances were removed (see Figure 10C). A negative lag time indicates that the trajectory was performed earlier in the cycle relative to the target trajectory.

**FIGURE 11:**
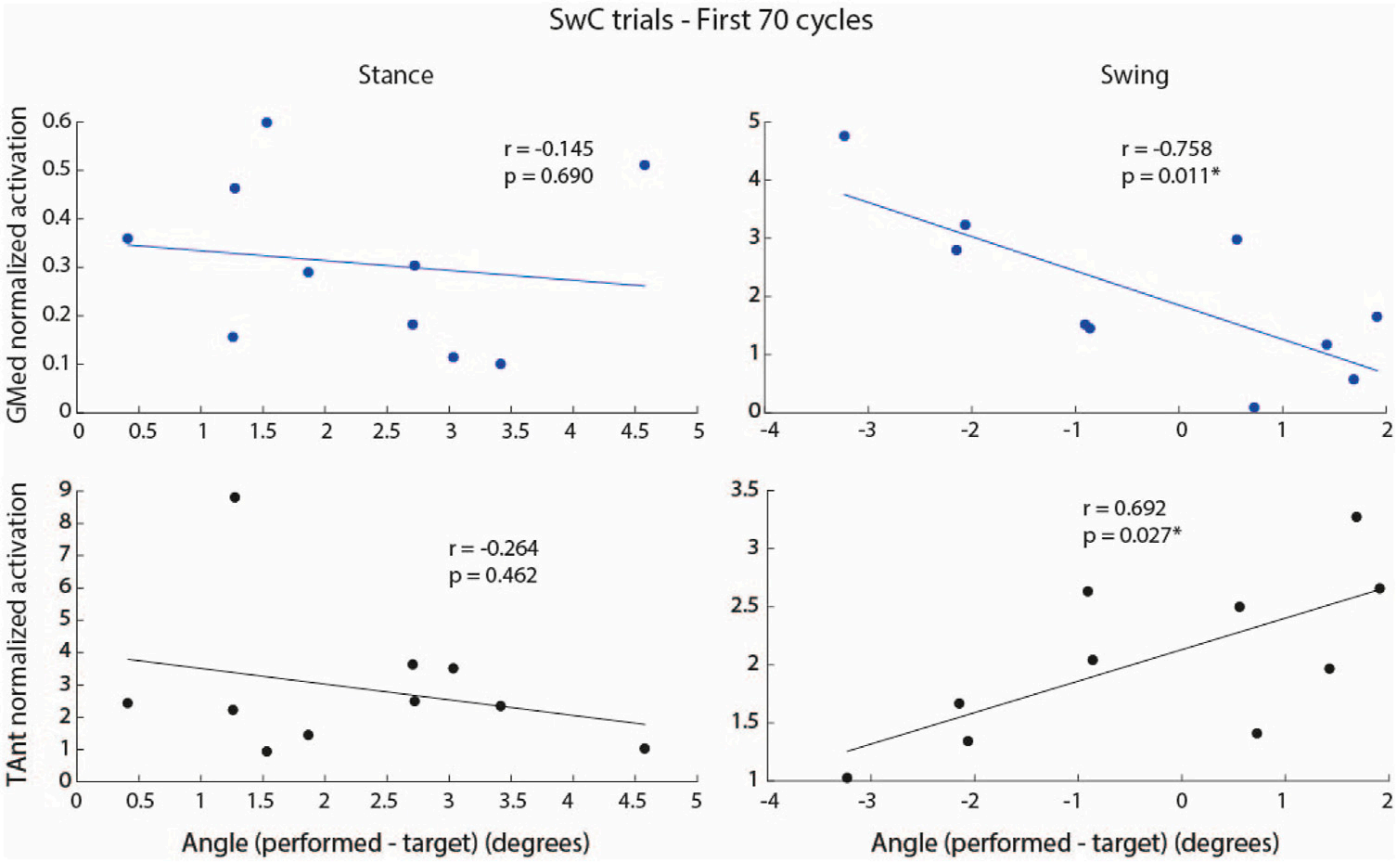
Correlation between integrated EMG activity of GMed (blue dots and regression lines) and TAnt (black dots and regression lines) and the error (mathematical subtraction) between maximum performed ankle angle and maximum target angle for stance and swing phases during the first 70 cycles of SwC trials. Each dot represents each subject. *p* < 0.05*.

Figure 11 shows the correlation between integrated EMG activity of ankle muscles (GMed and TAnt) and the error between maximum dorsiflexion angle performed and maximum target angle, for stance and swing. When SwC torque was applied (swing phase), subjects performed increased dorsiflexion as a result of decreased GMed activation (plantarflexor, negative correlation, r = −0.76, *p* < 0.05) and increased TAnt activation (dorsiflexor, positive correlation, r = 0.69, *p* < 0.05), *i.e.*, the lower the GMed activation and the higher the TAnt activation, the greater the dorsiflexion.

When SwC torque was not applied (stance phase), there were no significant correlations between the individual EMG activity of ankle muscles and the maximum ankle angle performed.

## 4 Discussion

Rehabilitation robotics such as MAFOs have the potential to facilitate the treatment of walking impairments in a wide spectrum of neurological disorders (Conner et al., 2021). MAFO-based therapies intend to leverage on the main principles of motor learning (e.g., repetition, task specificity, active engagement) (Conner et al., 2021). The novelty of this work consists in testing the hypothesis that adaptation of ankle joint muscles activity can be achieved through adaptive resistance applied by a MAFO in a standing still position condition. While there are several prior research works testing robot-mediated strategies during ankle mobilization or during walking (Zhang et al., 2013; Chin et al., 2017), targeting selectively the enhancement or adaptation of ankle muscles activation has not been tested, to the best of our knowledge, in the standing still position. From the clinical point of view, this condition may be beneficial during intermediate phases of rehabilitation between joint mobilization and gait training.

To improve current control algorithms and use robotic devices to drive beneficial neuroplasticity, it is fundamental not only to execute repetitive and intensive task-specific training (*e.g.*, gait training) (Krishnan et al., 2019) but also to understand muscle coordination changes and adaptations to robotic assistance so that these devices can be used in more natural and effective ways (Stampacchia et al., 2016; Jacobs et al., 2018; Barroso et al., 2019b).

In this study, we examined the adaptation of ankle muscles (GMed and TAnt) activity in response to the application of different ankle disturbances using a MAFO: a) dorsiflexion torque during the first part of the trajectory, with the shape of ground reaction force (StC disturbance), and b) plantarflexion torque during the second part of the trajectory (SwC disturbance). GMed activation decreased during the application of StC disturbance, indicating that dorsiflexion torque did not enhance GMed activity. We suggest that this might be explained by the unloaded body weight of the ipsilateral side, which might have decreased the activation of anti-gravity muscles. On the other hand, TAnt activation increased when SwC disturbance was applied, which indicates that plantarflexion torque succeeded in enhancing TAnt activation. Co-contraction was not induced for any disturbance paradigm, which would have been an undesired effect.

### 4.1 Muscle activity was similar across treadmill sessions

EMG envelopes of TAnt and GMed during treadmill trials were very similar for all subjects and were qualitatively similar to those described in the literature (Hidler and Wall, 2005; Barroso et al., 2014) (*i.e.*, TAnt was active from toe-off to mid-swing and from just before heel strike to mid-stance; GMed was active from the beginning of mid-stance until the end of terminal stance). The fact that, for each muscle, there were no statistically significant EMG activation differences across different treadmill trials suggests that treadmill sessions served as washout trials, which could exclude any neural effects on StC trials from previous SwC trials and *vice versa*.

### 4.2 Decreased gastrocnemius medialis activation when StC disturbance was applied

Our first hypothesis was not confirmed: GMed activity during stance was significantly lower during StC compared to baseline (Figure 6). When using a similar resistance approach to our StC paradigm, Conner et al. obtained increased plantarflexor activity and decreased dorsiflexor activity in individuals with cerebral palsy (Conner et al., 2020). However, they used a wearable ankle exoskeleton, with no weight load. Here, we hypothesize that having complete body load on the contralateral side and, consequently, unloaded body weight in the ipsilateral side leads to decreased GMed activation. Weight loading serves as an important afferent signal (Bastiaanse et al., 2000; Conner et al., 2020). Another possible interpretation for this decreased GMed activation is crossed inhibition of GMed motoneurons elicited by muscle afferents from the GMed of the standing (contralateral) leg. This inhibition of homonymous muscles from the contralateral side was already demonstrated in the Soleus (Mrachacz-Kersting et al., 2017). In our experiment, since the antigravity muscles of the standing leg (extensors, including GMed) were active to maintain upright position, this might have affected the overall excitability of the contralateral GMed: thus, Ia inhibitory interneurons in the contralateral spinal cord may explain, at least partially, the reduced activation.

### 4.3 Increased tibialis anterior activation when SwC disturbance was applied

Our first hypothesis was confirmed: TAnt presented significantly increased activity in SwC ON trials compared to baseline and POST SwC treadmill trials. We suggest that volitional engagement of TAnt to counteract SwC disturbances may have acted as a mechanical cue for the neuromuscular system to recruit additional motor units (Conner et al., 2020). Interestingly, TAnt activation also increased during stance (compared to baseline) when SwC was OFF. This may be due to permanent response to disturbances along each cycle, even though disturbances were applied during swing. The duration of each phase might not have been long enough to allow adaptation to specific phases, with and without disturbances within each cycle.

GMed activation was unchanged when SwC was on (swing phase), which was expected as additional plantarflexor activity was not necessary to counteract dorsiflexion torque. Interestingly, GMed activity significantly decreased during stance phase of SwC trials, when disturbances were not present. Based on the same observation of decreased GMed activity during the stance for StC trials described previously, we can also hypothesize that this is due to unloaded body weight of the ipsilateral side (Bastiaanse et al., 2000). As explained by Bastiaanse et al. (2000), unloaded treadmill walking significantly reduces EMG activity of GMed; in contrast, TAnt was even more active during unloading conditions.

Subjects learned to adapt ankle angle when SwC torque was applied (Figure 10). Immediately after removing SwC disturbances (SwC OFF 1 and SwC OFF 2), there were enhanced dorsiflexion peaks during swing, which indicates that subjects would have needed more than 16 cycles to re-adapt to MAFO training with no SwC disturbances. Adaptation during the 16 cycles after SwC removal consisted of anticipating dorsiflexion, probably as part of the previous strategy to adjust to the target trajectory when the effect was applied. Overall, results from Figure 10 indicate that subjects were able to follow the target trajectory when SwC torque was applied but were not able to follow the target trajectory immediately after removal of the SwC effect. Non-etheless, there is a trend in SwC OFF conditions indicating that users tend to perform increasingly similar trajectories to baseline with time.

Increased dorsiflexion during the swing phase of SwC trials may be due to decreased GMed activation, increased TAnt activation or a combination of both (Figure 11). However, we cannot determine whether the resultant angle was primarily performed as a function of TAnt activity alone, GMed activity alone or a combination of both. Due to the high activity levels, it seems that TAnt has a predominant role.

### 4.4 Limitations

One limitation of the MAFO was the maximum cadence that could be applied. Therefore, we decided to ask the participants to maintain the same cadence (30 cycles/minute) during treadmill and MAFO-based trials. This cadence was deemed as comfortable by all participants.

We also experienced technical difficulties to apply the StC paradigm, given the limitations of the technology to successfully apply dorsiflexion torques greater than 5 N⋅m. It is not clear whether this could have influenced GMed activation during the disturbance, *i.e.*, during the stance phase.

Muscular timing assessment is a common way to investigate gait dynamics (Di Nardo et al., 2019; Tigrini et al., 2020). This study could be complemented with an evaluation of the timing of activation of each muscle, for the different cycles, to investigate possible differences between the application and removal of disturbances. Here, we decided to compare muscle activity in terms of the amplitude of the EMG envelopes, for each phase of the cycle (stance and swing), which also allowed us to have a general picture of the activation timing of each muscle.

### 4.5 Future work

In the future, we intend to perform a longitudinal study with patients whose sensory and voluntary motor function is preserved, at least partially (e.g., hemiparetic stroke or incomplete spinal cord injury) aiming at simulating a gait-like feeling on the patients’ ankles, potentially triggering activity-dependent plasticity. In this sense, potential users need to meet strict inclusion criteria requirements that consider motor function scores and spasticity levels (*e.g.*, independent walking, passive ankle joint range at dorsiflexion of at least 90 degrees, dorsiflexor muscle strength of at least 2 according to Medical Research Council (MRC) index, ankle plantarflexor spasticity of ±2 according to Modified Ashworth Scale, among others (Asín-Prieto et al., 2020). Non-etheless, it is key to perform longer trial sessions in healthy volunteers first and assess (possible) electrophysiological changes after a longer training (e.g., changes in corticospinal excitability or neural drive).

As this was an exploratory study and the experimental protocol was long, we decided to explore the adaptation after removing the disturbances in SwC trials. Part of our future work may evolve around assessing adaptation when removing StC torque, as done for SwC. To achieve that goal, technical limitations such as the maximum dorsiflexion torque of 5 N⋅m need to be solved and different positions other than standing still (sitting, for example,) may also be explored.

This MAFO can also be combined with transcutaneous electrical nerve stimulation (TENS) to inhibit undesired sensorimotor activities (Kim et al., 2018), or functional electrical stimulation (FES) in order to guide and help adapt muscle activity (Barroso et al., 2019b), which would improve our platform towards recovery after neural injury.

## 5 Conclusion

We successfully tested novel ankle disturbances approaches that can be explored as potential resistance strategies in MAFO training. Results from SwC training warrant further investigation to promote specific motor recovery and learning of dorsiflexion in neural-impaired patients. This training can potentially be beneficial to foster increased volitional engagement of ankle dorsiflexors during intermediate phases of rehabilitation prior to overground exoskeleton-assisted training. Decreased activation of GMed during StC disturbance might be attributed to the unloaded body weight in the ipsilateral side, which typically decreases activation of anti-gravity muscles. Neural adaptation to StC disturbance will, therefore, be studied in different body postures in futures studies.

## Data Availability

The raw data supporting the conclusion of this article will be made available by the authors, upon reasonable request.

## Abbreviations

EMG, electromyography; FES, functional electrical stimulation; GMed, gastrocnemius medialis; MAFOs, motorized ankle foot orthoses; sEMG, surface electromyography; StC, stance correlate; SwC, swing correlate; TAnt, tibialis anterior; TENS, transcutaneous electrical nerve stimulation.

## References

[B1] Alessandro C. Barroso F. O. Prashara A. Tentler D. P. Yeh H.-Y. Tresch M. C. (2020). Coordination amongst quadriceps muscles suggests neural regulation of internal joint stresses, not simplification of task performance. Proc. Natl. Acad. Sci. 117, 8135–8142. 10.1073/pnas.1916578117 32205442 PMC7149390

[B2] Arazpour M. Bani M. A. Hutchins S. W. Curran S. Javanshir M. A. (2013). The influence of ankle joint mobility when using an orthosis on stability in patients with spinal cord injury: A pilot study. Spinal Cord. 51, 750–754. 10.1038/sc.2013.78 23896671

[B3] Asín-Prieto G. Martínez-Expósito A. Barroso F. O. Urendes E. J. Gonzalez-Vargas J. Alnajjar F. (2020). Haptic adaptive feedback to promote motor learning with a robotic ankle exoskeleton integrated with a video game. Front. Bioeng. Biotechnol. 8, 113. 10.3389/fbioe.2020.00113 32154239 PMC7047324

[B4] Barroso F. O. Alessandro C. Tresch M. C. (2019a). Adaptation of muscle activation after patellar loading demonstrates neural control of joint variables. Sci. Rep. 9, 20370–20412. 10.1038/s41598-019-56888-9 31889142 PMC6937258

[B5] Barroso F. O. Pascual-Valdunciel A. Torricelli D. Moreno J. C. Del Ama-Espinosa A. Laczko J. (2019b). “Noninvasive modalities used in spinal cord injury rehabilitation,” in Spinal cord injury therapy (London, UK: IntechOpen), 95–114.

[B6] Barroso F. O. Torricelli D. Molina-Rueda F. Alguacil-Diego I. M. Cano-de-la Cuerda R. Santos C. (2017). Combining muscle synergies and biomechanical analysis to assess gait in stroke patients. J. Biomechanics 63, 98–103. 10.1016/j.jbiomech.2017.08.006 28882330

[B7] Barroso F. O. Torricelli D. Moreno J. C. Taylor J. Gomez-Soriano J. Bravo-Esteban E. (2014). Shared muscle synergies in human walking and cycling. J. neurophysiology 112, 1984–1998. 10.1152/jn.00220.2014 25057144

[B8] Barroso F. Torricelli D. Moreno J. C. Taylor J. Gómez-Soriano J. Esteban E. B. (2013). “Similarity of muscle synergies in human walking and cycling: Preliminary results,” in 2013 35th annual international conference of the IEEE engineering in medicine and biology society (EMBC) (Osaka, Japan: IEEE), 6933–6936. 10.1109/EMBC.2013.6611152 24111339

[B9] Bastiaanse C. Duysens J. Dietz V. (2000). Modulation of cutaneous reflexes by load receptor input during human walking. Exp. Brain Res. 135, 189–198. 10.1007/s002210000511 11131503

[B10] Belda-Lois J.-M. Mena-del Horno S. Bermejo-Bosch I. Moreno J. C. Pons J. L. Farina D. (2011). Rehabilitation of gait after stroke: A review towards a top-down approach. J. neuroengineering rehabilitation 8, 66. 10.1186/1743-0003-8-66 PMC3261106 22165907

[B11] Ceseracciu E. Tagliapietra L. Moreno J. C. Asin G. del Ama A. J. Pérez S. (2017). “An emg-informed model to evaluate assistance of the biomot compliant ankle actuator,” in Wearable robotics: Challenges and trends (Berlin, Germany: Springer), 261–265.

[B12] Cheung V. C. Turolla A. Agostini M. Silvoni S. Bennis C. Kasi P. (2012). Muscle synergy patterns as physiological markers of motor cortical damage. Proc. Natl. Acad. Sci. 109, 14652–14656. 10.1073/pnas.1212056109 22908288 PMC3437897

[B13] Chin L. C. Basah S. N. Affandi M. Shah M. N. Yaacob S. Juan Y. E. (2017). Home-based ankle rehabilitation system: Literature review and evaluation. J. Teknol. 79. 10.11113/jt.v79.8468

[B14] Conner B. C. Luque J. Lerner Z. F. (2020). Adaptive ankle resistance from a wearable robotic device to improve muscle recruitment in cerebral palsy. Ann. Biomed. Eng. 48, 1309–1321. 10.1007/s10439-020-02454-8 31950309 PMC7096247

[B15] Conner B. C. Schwartz M. H. Lerner Z. F. (2021). Pilot evaluation of changes in motor control after wearable robotic resistance training in children with cerebral palsy. J. Biomechanics 126, 110601. 10.1016/j.jbiomech.2021.110601 PMC8453102 34332214

[B16] Di Nardo F. Strazza A. Mengarelli A. Cardarelli S. Tigrini A. Verdini F. (2019). Emg-based characterization of walking asymmetry in children with mild hemiplegic cerebral palsy. Biosensors 9, 82. 10.3390/bios9030082 31252517 PMC6784376

[B17] Ditunno J. Scivoletto G. (2009). Clinical relevance of gait research applied to clinical trials in spinal cord injury. Brain Res. Bull. 78, 35–42. 10.1016/j.brainresbull.2008.09.003 18848865

[B18] Eisinga R. Heskes T. Pelzer B. Te Grotenhuis M. (2017). Exact p-values for pairwise comparison of friedman rank sums, with application to comparing classifiers. BMC Bioinforma. 18, 68. 10.1186/s12859-017-1486-2 PMC5267387 28122501

[B19] Hermens H. J. Freriks B. Merletti R. Stegeman D. Blok J. Rau G. (1999). European recommendations for surface electromyography. Roessingh Res. Dev. 8, 13–54.

[B20] Hidler J. M. Wall A. E. (2005). Alterations in muscle activation patterns during robotic-assisted walking. Clin. Biomech. 20, 184–193. 10.1016/j.clinbiomech.2004.09.016 15621324

[B21] Hug F. Turpin N. A. Couturier A. Dorel S. (2011). Consistency of muscle synergies during pedaling across different mechanical constraints. J. neurophysiology 106, 91–103. 10.1152/jn.01096.2010 21490282

[B22] Jacobs D. A. Koller J. R. Steele K. M. Ferris D. P. (2018). Motor modules during adaptation to walking in a powered ankle exoskeleton. J. neuroengineering rehabilitation 15, 2. 10.1186/s12984-017-0343-x PMC5751608 29298705

[B23] Kaczmarek P. Mańkowski T. Tomczyński J. (2019). putemg—a surface electromyography hand gesture recognition dataset. Sensors 19, 3548. 10.3390/s19163548 31416251 PMC6720505

[B24] Kim Y. Cho H.-J. Park H.-S. (2018). Technical development of transcutaneous electrical nerve inhibition using medium-frequency alternating current. J. neuroengineering rehabilitation 15, 80. 10.1186/s12984-018-0421-8 PMC6102860 30126438

[B25] Krishnan C. Dharia A. K. Augenstein T. E. Washabaugh E. P. Reid C. E. Brown S. R. (2019). Learning new gait patterns is enhanced by specificity of training rather than progression of task difficulty. J. biomechanics 88, 33–37. 10.1016/j.jbiomech.2019.03.014 PMC6512968 30905405

[B26] Moltedo M. Baček T. Verstraten T. Rodriguez-Guerrero C. Vanderborght B. Lefeber D. (2018). Powered ankle-foot orthoses: The effects of the assistance on healthy and impaired users while walking. J. neuroengineering rehabilitation 15, 86. 10.1186/s12984-018-0424-5 PMC6167899 30285869

[B27] Moreno J. C. Barroso F. Farina D. Gizzi L. Santos C. Molinari M. (2013). Effects of robotic guidance on the coordination of locomotion. J. neuroengineering rehabilitation 10, 79. 10.1186/1743-0003-10-79 PMC3724716 23870328

[B28] Mrachacz-Kersting N. Geertsen S. S. Stevenson A. J. T. Nielsen J. B. (2017). Convergence of ipsi-and contralateral muscle afferents on common interneurons mediating reciprocal inhibition of ankle plantarflexors in humans. Exp. Brain Res. 235, 1555–1564. 10.1007/s00221-016-4871-6 28258435

[B29] Paul L. Brewster S. Wyke S. Gill J. M. Alexander G. Dybus A. (2016). Physical activity profiles and sedentary behaviour in people following stroke: A cross-sectional study. Disabil. rehabilitation 38, 362–367. 10.3109/09638288.2015.1041615 25936730

[B30] Pongpipatpaiboon K. Mukaino M. Matsuda F. Ohtsuka K. Tanikawa H. Yamada J. (2018). The impact of ankle–foot orthoses on toe clearance strategy in hemiparetic gait: A cross-sectional study. J. neuroengineering rehabilitation 15, 41. 10.1186/s12984-018-0382-y PMC5966858 29792211

[B31] Safavynia S. Torres-Oviedo G. Ting L. (2011). Muscle synergies: Implications for clinical evaluation and rehabilitation of movement. Top. spinal cord Inj. rehabilitation 17, 16–24. 10.1310/sci1701-16 PMC3143193 21796239

[B32] Shi B. Chen X. Yue Z. Yin S. Weng Q. Zhang X. (2019). Wearable ankle robots in post-stroke rehabilitation of gait: A systematic review. Front. neurorobotics 13, 63. 10.3389/fnbot.2019.00063 PMC6700322 31456681

[B33] Stampacchia G. Rustici A. Bigazzi S. Gerini A. Tombini T. Mazzoleni S. (2016). Walking with a powered robotic exoskeleton: Subjective experience, spasticity and pain in spinal cord injured persons. NeuroRehabilitation 39, 277–283. 10.3233/nre-161358 27372363

[B34] Takahashi K. Z. Lewek M. D. Sawicki G. S. (2015). A neuromechanics-based powered ankle exoskeleton to assist walking post-stroke: A feasibility study. J. neuroengineering rehabilitation 12, 23. 10.1186/s12984-015-0015-7 PMC4367918 25889283

[B35] Tigrini A. Mengarelli A. Cardarelli S. Fioretti S. Verdini F. (2020). Improving emg signal change point detection for low snr by using extended teager-kaiser energy operator. IEEE Trans. Med. Robotics Bionics 2, 661–669. 10.1109/tmrb.2020.3014517

[B36] Tudor-Locke C. Camhi S. M. Leonardi C. Johnson W. D. Katzmarzyk P. T. Earnest C. P. (2011). Patterns of adult stepping cadence in the 2005–2006 nhanes. Prev. Med. 53, 178–181. 10.1016/j.ypmed.2011.06.004 21708187

[B37] Van Der Salm A. Nene A. V. Maxwell D. J. Veltink P. H. Hermens H. J. Ijzerman M. J. (2005). Gait impairments in a group of patients with incomplete spinal cord injury and their relevance regarding therapeutic approaches using functional electrical stimulation. Artif. organs 29, 8–14. 10.1111/j.1525-1594.2004.29004.x 15644078

[B38] Winstein C. Lewthwaite R. Blanton S. R. Wolf L. B. Wishart L. (2014). Infusing motor learning research into neurorehabilitation practice: A historical perspective with case exemplar from the accelerated skill acquisition program. J. neurologic Phys. Ther. JNPT 38, 190–200. 10.1097/npt.0000000000000046 PMC5348298 24828523

[B39] Winter D. A. (1991). Biomechanics and motor control of human gait: Normal, elderly and pathological. Waterloo, ON: University of Waterloo Press.

[B40] Yeung L.-F. Ockenfeld C. Pang M.-K. Wai H.-W. Soo O.-Y. Li S.-W. (2018). Randomized controlled trial of robot-assisted gait training with dorsiflexion assistance on chronic stroke patients wearing ankle-foot-orthosis. J. neuroengineering rehabilitation 15, 51. 10.1186/s12984-018-0394-7 PMC6006663 29914523

[B41] Zehr E. P. (2011). Evidence-based risk assessment and recommendations for physical activity clearance: Stroke and spinal cord injury^1^This paper is one of a selection of papers published in this special issue, entitled evidence-based risk assessment and recommendations for physical activity clearance, and has undergone the journal’s usual peer review process. Appl. Physiology, Nutr. Metabolism 36, S214–S231. 10.1139/h11-055 21800943

[B42] Zhang M. Davies T. C. Xie S. (2013). Effectiveness of robot-assisted therapy on ankle rehabilitation–a systematic review. J. neuroengineering rehabilitation 10, 30–16. 10.1186/1743-0003-10-30 PMC3636117 23517734

